# Supplementary material for: Saccharomyces boulardii (CNCM I-745) improves intestinal damage in sepsis by remodeling intestinal flora structure
Source: Front Cell Infect Microbiol. 2025 Jul 31;15:1602792. doi: 10.3389/fcimb.2025.1602792 (PMC12350329; doi:10.3389/fcimb.2025.1602792)
Supplement: Supplementary file 1 [file DataSheet1.zip › summary/summary/8_Advanced_analyse/2_PICRUSt2_result/METACYC/Group.BW4_vs_AW4/PICRUSt2_METACYC_Group.BW4_vs_AW4_diff.pdf]

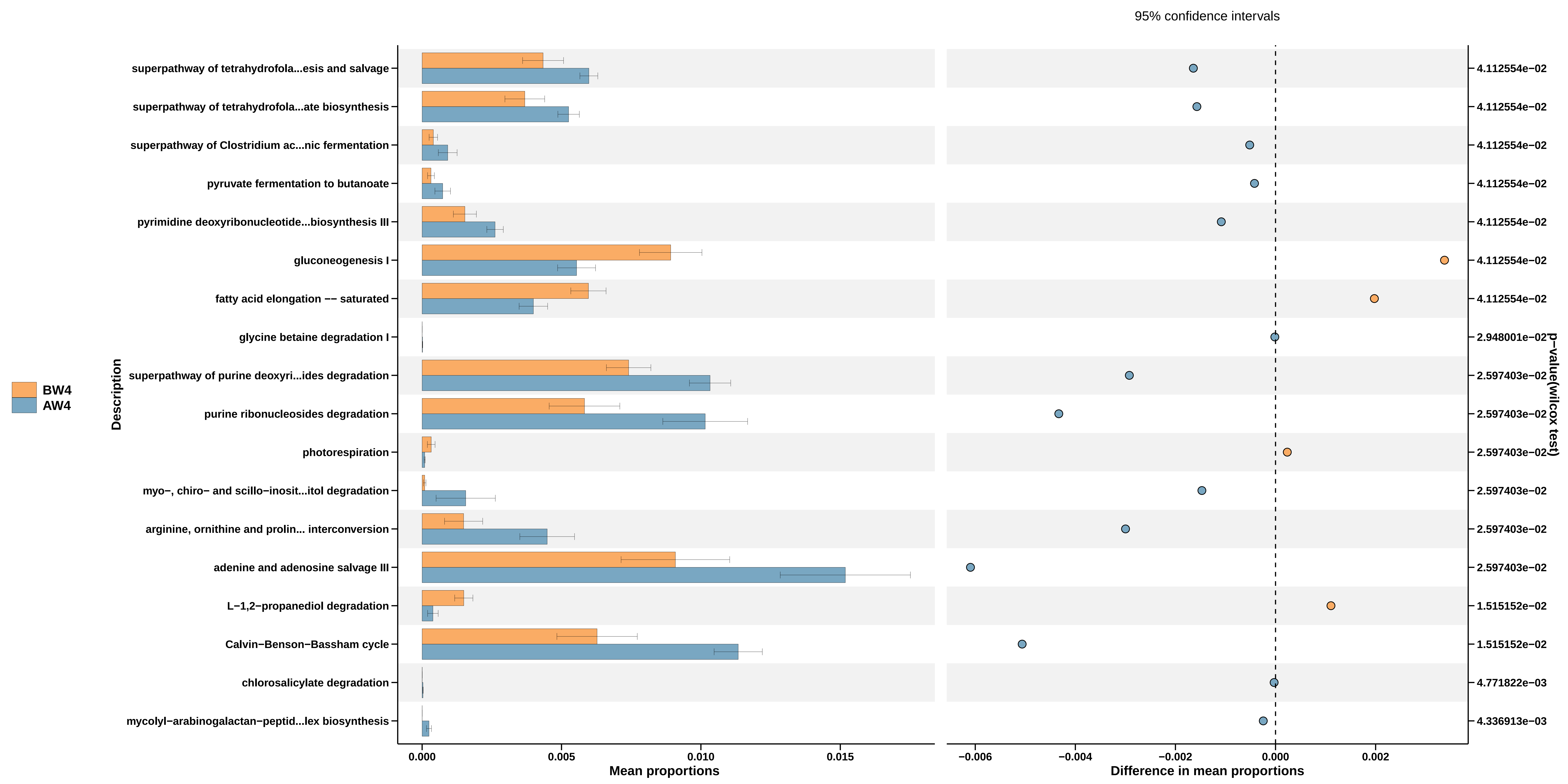
95% confidence intervals
BW4
AW4
Description
superpathway of tetrahydrofola...esis and salvage
superpathway of tetrahydrofola...ate biosynthesis
superpathway of Clostridium ac...nic fermentation
pyruvate fermentation to butanoate
pyrimidine deoxyribonucleotide...biosynthesis III
gluconeogenesis I
fatty acid elongation −− saturated
glycine betaine degradation I
superpathway of purine deoxyri...ides degradation
purine ribonucleosides degradation
photorespiration
myo−, chiro− and scillo−inosit...itol degradation
arginine, ornithine and prolin... interconversion
adenine and adenosine salvage III
L−1,2−propanediol degradation
Calvin−Benson−Bassham cycle
chlorosalicylate degradation
mycolyl−arabinogalactan−peptid...lex biosynthesis
0.000
0.005
0.010
0.015
Mean proportions
−0.006
−0.004
−0.002
0.000
0.002
Difference in mean proportions
p−value(wilcox test)
4.112554e−02
4.112554e−02
4.112554e−02
4.112554e−02
4.112554e−02
4.112554e−02
4.112554e−02
2.948001e−02
2.597403e−02
2.597403e−02
2.597403e−02
2.597403e−02
2.597403e−02
2.597403e−02
1.515152e−02
1.515152e−02
4.771822e−03
4.336913e−03
